# Radiographic imaging for traumatic ankle injuries: a demand profile and investigation of radiological reporting timeframes from an Australian tertiary facility

**DOI:** 10.1186/1757-1146-7-25

**Published:** 2014-05-03

**Authors:** Patrick Eastgate, Robert Davidson, Steven M McPhail

**Affiliations:** 1 Royal Brisbane and Women’s Hospital, Butterfield Street, Herston, Brisbane 4029, Australia; 2 Discipline of Medical Radiation Science, School of Dentistry and Health Sciences, Charles Sturt University, Wagga Wagga 2678, Australia; 3 Centre for Functioning and Health Research, Queensland Health, Cnr of Ipswich Road and Cornwall Street, Brisbane, Australia; 4 School of Public Health and Social Work and Institute of Health and Biomedical Innovation, Queensland University of Technology, Victoria Park Road, Brisbane, Australia

**Keywords:** Ankle, Radiology, Radiograph, Reporting, Demand, X-ray, Emergency, Trauma, Orthopaedic, Fracture

## Abstract

**Background:**

Radiographic examinations of the ankle are important in the clinical management of ankle injuries in hospital emergency departments. National (Australian) Emergency Access Targets (NEAT) stipulate that 90 percent of presentations should leave the emergency department within 4 hours. For a radiological report to have clinical usefulness and relevance to clinical teams treating patients with ankle injuries in emergency departments, the report would need to be prepared and available to the clinical team within the NEAT 4 hour timeframe; before the patient has left the emergency department. However, little is known about the demand profile of ankle injuries requiring radiographic examination or time until radiological reports are available for this clinical group in Australian public hospital emergency settings.

**Methods:**

This study utilised a prospective cohort of consecutive cases of ankle examinations from patients (n = 437) with suspected traumatic ankle injuries presenting to the emergency department of a tertiary hospital facility. Time stamps from the hospital Picture Archiving and Communication System were used to record the timing of three processing milestones for each patient’s radiographic examination; the time of image acquisition, time of a provisional radiological report being made available for viewing by referring clinical teams, and time of final verification of radiological report.

**Results:**

Radiological reports and all three time stamps were available for 431 (98.6%) cases and were included in analysis. The total time between image acquisition and final radiological report verification exceeded 4 hours for 404 (92.5%) cases. The peak demand for radiographic examination of ankles was on weekend days, and in the afternoon and evening. The majority of examinations were provisionally reported and verified during weekday daytime shift hours.

**Conclusions:**

Provisional or final radiological reports were frequently not available within 4 hours of image acquisition among this sample. Effective and cost-efficient strategies to improve the support provided to referring clinical teams from medical imaging departments may enhance emergency care interventions for people presenting to emergency departments with ankle injuries; particularly those with imaging findings that may be challenging for junior clinical staff to interpret without a definitive radiological report.

## Background

The unique anatomy of the ankle and its functional relationship with the foot make it highly susceptible to injury [1-5]. Ankle injuries are one of the most common injuries presenting to the emergency setting and may involve fractures, dislocations and ligamentous injuries [6-8]. It has been reported that between 5–12% of all patients who attend the emergency department involve an ankle injury [7-9]. Radiographs of the ankle joint make up to 10% of all radiographic examinations in the emergency setting [6,10].

Missed and or delayed diagnoses in the emergency department can have severe consequences and are a major patient safety concern [11,12]. Emergency departments often have inexperienced junior doctors seeing large numbers of patients of all ages every day; errors or missed diagnoses do occur [13,14]. The perception that ankle fractures have a low rate of sub-optimal outcome and negligible negative long term consequence are not founded in empirical data, with life impacts following ankle fractures potentially extending into a range of life domains beyond physical discomfort [15,16]. The severity of life impact may not necessarily be dependent on the severity of the initial trauma, but sub-optimal clinical management could contribute to chronic and persistent conditions [15,17].

In Australia, public hospital emergency departments are now required to meet the National Emergency Access Target (NEAT). The objective and output of this schedule requires that within 4 hours, 90 percent of all patients presenting to a public hospital emergency department will either physically leave the emergency department for admission to hospital, be referred to another hospital for treatment, or be discharged from hospital [18]. For a radiological report to have clinical usefulness and relevance to clinical teams treating patients with ankle injuries in emergency departments, the report would need to be prepared and available to the clinical team within the NEAT 4 hour timeframe; before the patient has left the emergency department. Therefore, a 4 hour limit could be used as a conservative estimate for the provision of a radiological report within a clinically relevant timeframe if it were intended to influence the referring clinical teams in emergency departments.

Junior clinical staff in the emergency setting, and their patients, will likely benefit from definitive radiological reporting on ankle radiographs within clinically relevant timeframes. The provision of an accurate radiological report provides junior doctors with clear information to help them make appropriate and timely clinical decisions. However, radiologists in emergency hospital settings are faced with service delivery pressures [19] and possible delays in the reporting of (non-life threatening) ankle injuries in emergency settings may occur. Delay in radiological reporting on traumatic ankle injuries may increase the risk of diagnostic error, compromise patient safety and potentially lead to malpractice claims [11]. However, little is known about the demand profile of ankle injuries requiring radiographic examination or time until radiological reports are available for this clinical group in Australian public hospital emergency settings.

The aim of this investigation was to describe the demand profile for radiological ankle examinations in a tertiary emergency department setting and investigate the process times from radiographic image capture to radiological report becoming available to the referring clinical team. Specifically, the study investigated the frequency of ankle radiological examination (by day of week and time of day), as well as the time between three process milestones; image capture, provisional report; and verification of the final radiological report.

## Methods

### Design

This study utilised a prospective cohort of consecutive cases.

### Ethical statement

This investigation was approved by the Royal Brisbane and Women’s Hospital Human Research Ethics Committee and complied with the Declaration of Helsinki.

### Participants and setting

Radiographic ankle examinations were from patients with suspected traumatic ankle injuries presenting to the Emergency department of a tertiary hospital facility in Brisbane, Australia. The medical imaging service for this emergency department operates 24 hours per day, everyday. Medical imaging staffing in this unit is based on a three shift per day schedule; day (08:00–16:00), late (16:00–00:00) and night (00:00–08:00). All cases referred for radiographic examinations for suspected traumatic ankle injury were tracked for 12 weeks (n = 437). Cases that included concurrent investigations for other body regions (for example, the foot or knee) in addition to a suspected ankle injury were included to ensure that the dataset was reflective of usual practice; not just the simplest cases. There were no exclusion criteria. Radiological reports were either provisionally completed by radiology registrars (typically with one to four years experience), then later verified by a consultant radiologist, or were reported and verified directly by a consultant radiologist.

### Demand profile measures collected

The age of the patient and side of suspected injury was recorded for each patient. To describe the demand profile for radiographic imaging from suspected traumatic ankle injuries, three key dates and times were recorded for each case. This included the time and date that the radiographic ankle examinations were taken, the time the electronic provisional radiological report was completed, and the time that the radiological report was electronically verified by a consultant radiologist on the Picture Archiving Communication System. In this context, the provisional report refers to when a radiological report has been dictated by a registrar or consultant but not yet reviewed in its typed format on the Picture Archiving Communication System. The typed provisional report can be accessed by clinical teams prior to verification by a consultant radiologist. The electronic radiological report verification occurred when a consultant radiologist had reviewed the provisional report on the Picture Archiving Communication System and confirmed its accuracy (or completed the necessary modifications to ensure the report was accurate).

### Procedure

Consecutive patient cases presenting to the emergency department that met inclusion criteria were identified by the request code for radiographic examination for suspected ankle trauma for each day of the study period. This included requests for a radiographic ankle series to be undertaken in the emergency department (trauma) setting; but did not include cases with suspected trauma requested from other hospital units or non-trauma settings. Demand profile measures were collected directly from the hospital’s computerised medical imaging management system which time-stamped the point at which each of the three key events occurred. This information was collated by a member of the research team (PE) and prepared for analysis. To prepare the data for analysis to describe the demand profile, two additional codes were added for each date and time point. First, the day of the week (Monday to Sunday) that the event occurred was coded (one to seven). Second, the time of day that the event occurred was coded into 1 of 6, 4 hour blocks. These 4 hour blocks coincided with the first and second halves of the three (8 hour) shifts worked by medical imaging staff in this unit.

### Analysis

The mean (standard deviation) age of patients and number (percentage) of left and right sided examinations were calculated to describe the patient sample. To describe the time-of-day demand, frequency histograms were used to present the number of: radiographic examinations completed per day-of-week and 4-hour time block; provisional radiological reports completed by day-of-week and 4-hour time block; and radiological reports verified by day-of-week and 4-hour time block. The mean (standard error) delay between radiographic image acquisition and provisional radiological report completion was presented in frequency histograms per day-of-week and 4-hour time block. The mean (standard error) delay between provisional radiological reporting and verification of final radiological report were presented in frequency histograms per day-of-week and 4-hour time block. Similarly, the mean (standard error) delay between radiographic image acquisition and verification of final radiological report was presented in frequency histograms per day-of-week and 4-hour time block.

## Results

The mean (standard deviation) age of patients included in the sample was 38.0 (18.6) years. Of the 437 consecutive ankle cases included in this investigation, 211 were left ankles and 226 were right ankles. Radiological reports were completed for 431 (98.6%) cases and were included in the analyses. The mean time from image acquisition to provisional radiological report was 31.9 hours; with the time between image acquisition and provisional radiological report exceeding 4 hours for 360 (82.4%) cases. The mean total time between image acquisition and final radiological report verification was 84.0 hours; with the time between image acquisition and final radiological report exceeding 4 hours for 404 (92.5%) cases.

**Figure 1:**
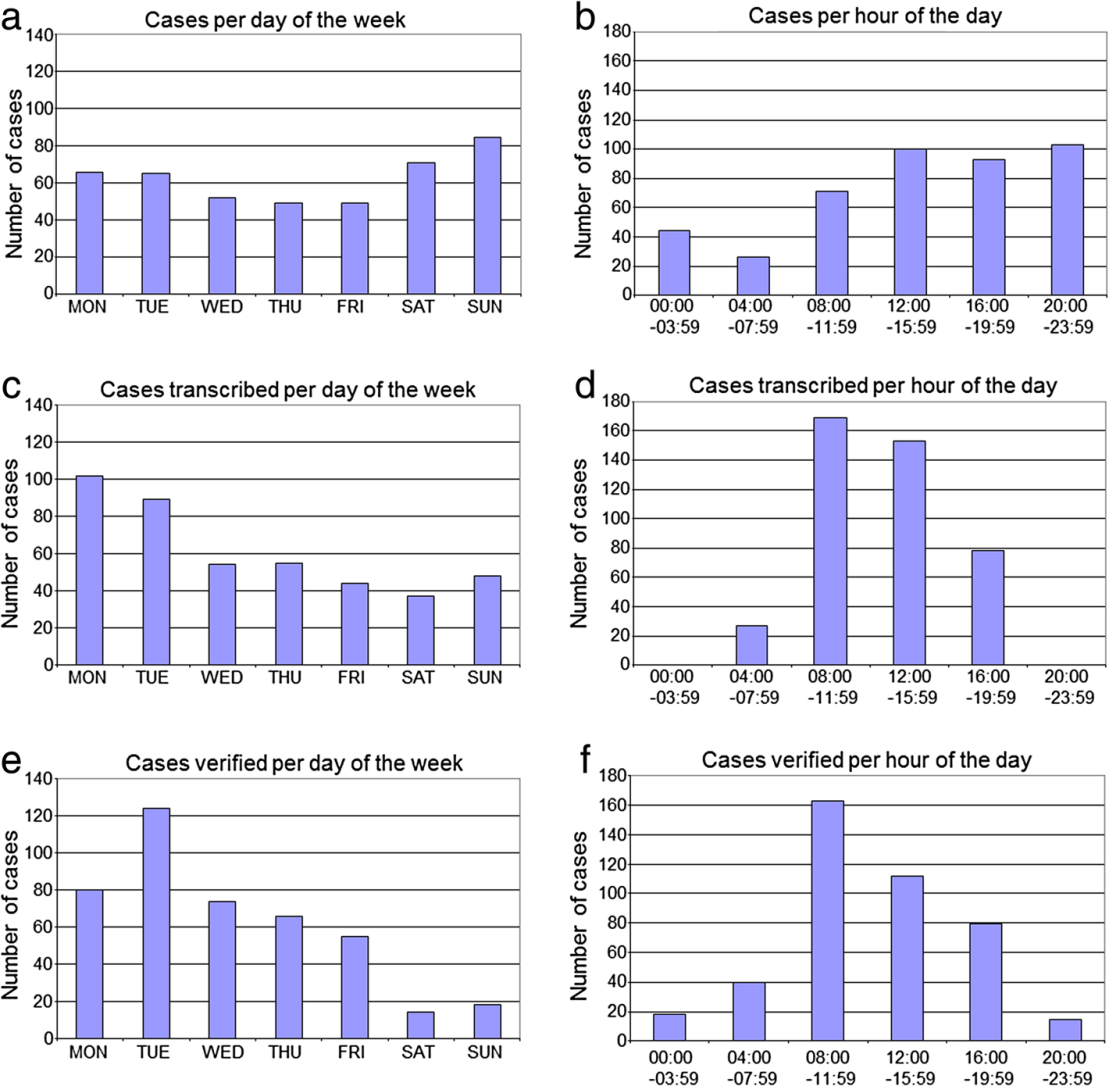
Frequency of radiographic image capture per day of week (a) and time of day (b), provisional report completed by day of week (c) and time of day (d), and radiological report verification by day of week (e) and time of day (f).

The day-of-week and time-of-day demand frequency histograms are presented in Figure 1. The demand for radiographic ankle examinations peaked on weekend days (Figure 1a), and in the afternoon and evening (Figure 1b). The number of provisional radiological reports completed peaked on Mondays and Tuesdays with fewer provisional reports completed from Wednesday to Sunday (Figure 1c). The majority of provisional reports were completed during the day shift hours (Figure 1d). The number of radiological report verifications peaked on Tuesdays (Figure 1e); with the majority of report verifications completed during the day shift (Figure 1f).

**Figure 2:**
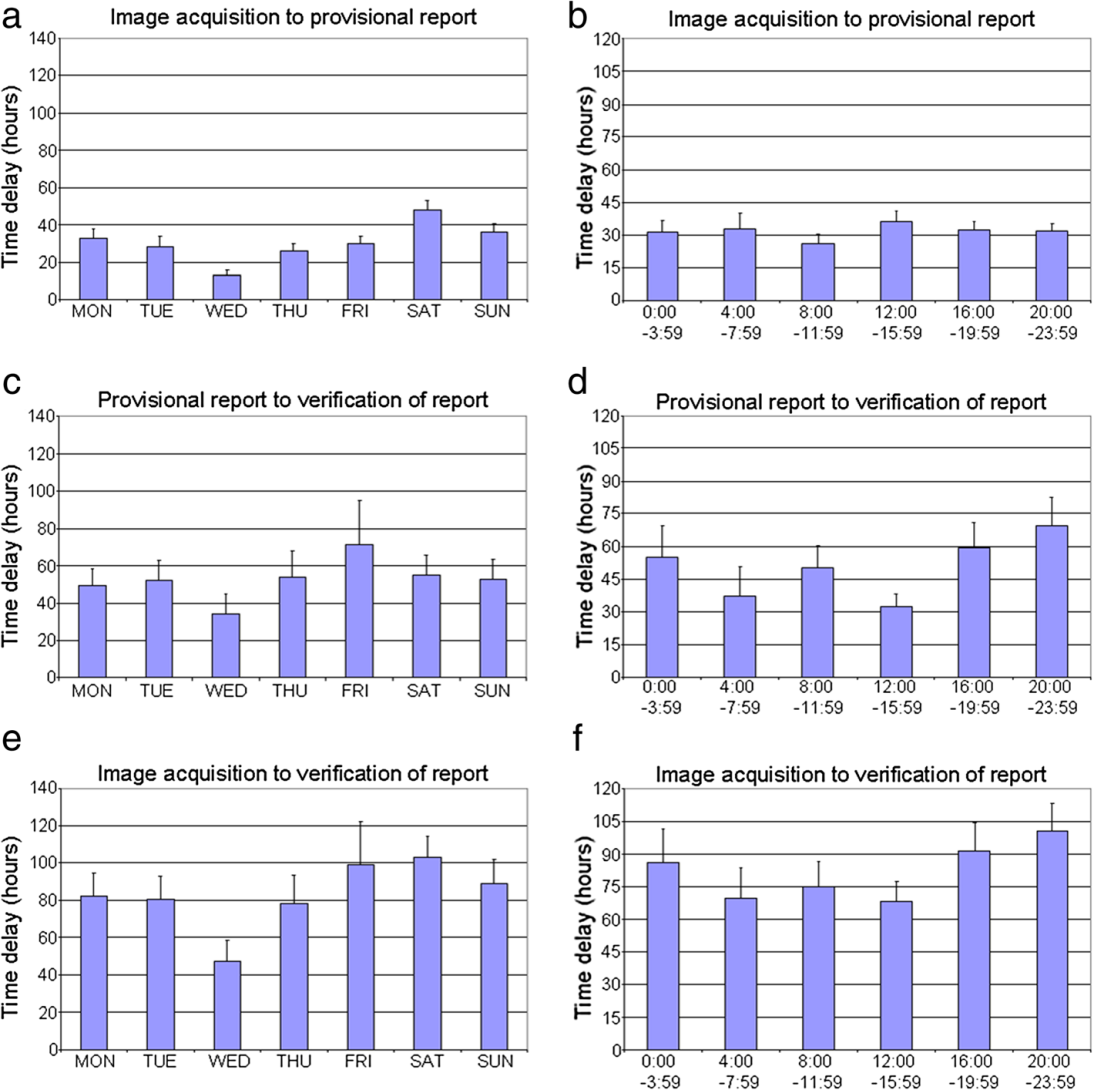
Time delay in hours from radiographic image capture to provisional report per day of week (a) and time of day (b), from provisional report to report verification per day of week (c) and time of day (d), and from radiographic image capture to report verification per day of week (e) and time of day (f).

The mean (and standard error) delay between events are presented in Figure 2 per day-of-week and time-of-day that the radiographic image was acquired. The mean time from image acquisition to provisional radiological report exceeded 24 hours for all days of the week except images acquired on Wednesdays; only 77 (17.6%) of provisional reports for all images were completed within 4 hours of image acquisition. The mean time from image acquisition to radiological report verification exceeded four days for images acquired on Fridays and Saturdays; and exceeded three days for Mondays, Tuesdays and Thursdays. Images acquired on Wednesdays had the shortest delays between image acquisition and report verification. Images acquired between 04:00 and 16:00 had the shortest delays between image acquisition and report verification.

## Discussion

This study indicated that radiographic examinations of the ankle were common on all days of the week; although most frequent from Saturday through to Tuesday with peak demand on Sundays. This peak in demand may be attributed to increased recreational activity on weekends; including sporting and social activities or perhaps the consumption of alcohol. In contrast, the frequency of radiological reporting was highest at the start of the weekdays, particularly Monday and Tuesday, with a clear reduction in reporting over the weekend. Verification of reports peaked on Tuesdays with fewer verifications occurring over the weekend days. This would be consistent with fewer senior radiology registrars and radiology consultants available to review radiographic imaging on weekend days. While demand for radiographic examinations of the ankle occurred throughout the 24 hour cycle, demand peaked between midday and midnight. On the other hand, reporting predominantly occurred during business hours from 08:00 until 16:00.

In the setting of the present study, the length of time between image capture and radiological reporting was less than ideal. The mean delay between image capture and provisional radiological reporting exceed 24 hours on most days; with images captured on Friday and Saturday experiencing the longest delay from image capture to verification of the final report. This was most likely due to a backlog of images accumulating over the weekends that were not signed off until business hours the following week. In contemporary emergency settings, it would seem likely that patient treatment plans would have been implemented within 24 hours for most (or all) patients.

The delay from image capture to any form of radiological opinion being available within a useful clinical timeframe may have resulted in junior referring clinicians interpreting radiographic ankle images and making treatment decisions without radiological opinion. In the emergency setting, many of these decisions would have involved the patient being discharged home before a radiological report was available. While the referring clinicians have undoubtedly completed appropriate qualifications and are competent in their practice, it is plausible that a delay in radiographic opinion may lead to an increase risk to the patient being discharged with unseen pathology or inappropriate treatment and follow up. One of the conditions where this is possible includes fractures of the lateral process of the talus. These fractures may be difficult to recognise on plain film radiographs and can result in secondary osteoarthritis of the ankle or talo-calcaneal joints, chronic pain and stiffness [20].

Prior research in hospital settings has identified the potential existence of a ‘weekend effect’; whereby patients admitted to hospitals on weekends experienced slightly higher risk-adjusted mortality than patients that were admitted on weekdays [21]. This weekend effect was observed to be larger in major teaching hospitals and should be a cause for concern. The present study has provided some empirical data demonstrating that all days of the week are not necessarily equal for patients presenting with traumatic ankle injuries. Avoiding delays between capture of radiographic imaging and availability of a radiographic comment may reduce the risk of missed or incorrect diagnoses.

Any potential solution to overcoming delays between image capture and radiological reporting should involve human resource considerations. Medical imaging teams providing services in emergency settings may need to account for increasing demand at peak times. Concealed within this seemingly straightforward suggestion of matching staffing to demand sits a host of complexity associated with potential human resource issues. These issues may include greater labour costs associated with weekend and after hours work, as well as industrial relation agreements surrounding shift work conditions and entitlements. Although the detail of these issues extend beyond the scope of the present study, the findings from this investigation have highlighted that peak demand for ankle fracture imaging does not occur during weekday business hours. With contemporary communication technologies permitting rapid transfer of high resolution imaging data in real time, use of radiologists in remote-locations offers another potential option for workforce flexibility to meet fluctuating demand on medical imaging services.

Ankle fractures are just one of many traumatic conditions that place demand on medical imaging services in emergency settings, other traumatic presentations may (or may not) follow and contribute the same pattern of delays. Further examination of demand for emergency medical imaging services for other traumatic conditions are worthy of consideration. Additionally, future research could also consider not only the demand for services, but also patient outcomes (particularly adverse outcomes that may be associated with missed or incorrect diagnoses).

There were strengths and limitations associated with this research design. First, the use of consecutive cases could be considered a strength of the study. A second strength was the collection of data directly from the Picture Archiving and Communication System being used by the medical imaging department. This ensured that the collected date and time data accurately represented the capture of images and availability of reports in the clinical setting for a large sample of patients. However, the associated limitation with using this system was the inability of the investigators to capture further clinical details, including patient outcomes. Another limitation was that this investigation included a tertiary hospital in a developed nation. The demand profile of dissimilar health services may not be congruent with findings reported in this investigation. For example, smaller hospitals may experience lesser or greater delays than those observed in this investigation.

## Conclusions

This investigation was successful in addressing its aim to describe the demand profile for radiological ankle examinations in a tertiary emergency department setting and investigated the process times from radiographic image capture to radiological report available to the referring clinical team. The demand profile and image reporting time-frames were not equal across the days of week or time of day. Demand for radiographic images of the ankle peaked from Saturday to Tuesday. Radiographic images taken on Friday and Saturday experienced the longest delay from image capture to verification of final radiological report. In the present study, radiological opinion was frequently not available within clinically useful timeframes. This may contribute to the risk of missed or incorrect diagnoses and clinical management; however, further research including patient outcomes is warranted.

## Competing interests

The authors declare that they have no competing interests.

## Authors’ contributions

PE and SMM participated in the design of the study and performed the analyses. PE and SMM conceived of the study, and participated in its design, coordination and principle drafting of the manuscript. RD participated in appraisal and editing of the manuscript. All authors read and approved the final manuscript.
